# Differences between the dispatch priority assessments of emergency medical dispatchers and emergency medical services: a prospective register-based study in Finland

**DOI:** 10.1186/s13049-023-01072-2

**Published:** 2023-02-16

**Authors:** Tomi Salminen, Kaius Kaartinen, Mervi Roos, Verna Vaajanen, Ari Ekstrand, Piritta Setälä, Sanna Hoppu

**Affiliations:** 1 Tampere University of Applied Sciences, Kuntokatu 3, 33520 Tampere, Finland; 2 Centre for Prehospital Emergency Care, Emergency Medical Services, Wellbeing Services County of Pirkanmaa, Satakunnankatu 16, 33100 Tampere, Finland; 3 Health Sciences Unit, Faculty of Social Sciences, Tampere University, Arvo Ylpön Katu 34, 33520 Tampere, Finland; 4 Faculty of Medicine and Health Technology, Tampere University, Arvo Ylpön Katu 34, 33520 Tampere, Finland; 5 Emergency Response Centre Agency, P.O. Box 112, 28131 Pori, Finland

**Keywords:** Ambulance, Emergency Medical Communication Centre, Emergency medical dispatch, Emergency medical services, Pre-hospital triage, Telephone triage

## Abstract

**Background:**

Responsive and efficient emergency medical services (EMS) require accurate telephone triage. In Finland, such services are provided by Emergency Response Centre Agency (ERC Agency). In 2018, a new Finnish computer-assisted emergency dispatch system was introduced: the Emergency Response Integrated Common Authorities (ERICA). After the introduction of ERICA, the appropriateness of EMS dispatch has not been investigated yet. The study´s objective is to determine the consistency between the priority triage of the emergency medical dispatcher (EMD) and the on-scene priority assessment of the EMS, and whether the priority assessment consistency varied among the dispatch categories.

**Methods:**

This was a prospective register-based study. All EMS dispatches registered in the Tampere University Hospital area from 1 August 2021 to 31 August 2021 were analysed. The EMD’s mission priority triaged during the emergency call was compared with the on-scene EMS’s assessment of the priority, derived from the pre-set criteria. The test performance levels were measured from the crosstabulation of true or false positive and negative values of the priority assessment. Statistical significance was analysed using the chi-square test and the Kruskal–Wallis H test, and *p*-values < 0.05 were considered significant.

**Results:**

Of the 6416 EMS dispatches analysed in this study, 36% (2341) were urgent according to the EMD’s dispatch priority, and of these, only 29% (688) were urgent according to the EMS criteria. On the other hand, 64% (4075) of the dispatches were non-urgent according to the EMD’s dispatch priority, of which 97% (3949) were non-urgent according to the EMS criteria. Moreover, there were differences between the EMD and EMS priority assessments among the dispatch categories (*p* < 0.001). The overall efficiency was 72%, sensitivity 85%, specificity 71%, positive predictive value 29%, and negative predictive value 97%.

**Conclusion:**

While the EMD recognised the non-urgent dispatches with high consistency with the EMS criteria, most of the EMD’s urgent dispatches were not urgent according to the same criteria. This may diminish the availability of the EMS for more urgent missions. Thus, measures are needed to ensure more accurate and therefore, more efficient use of EMS resources in the future.

**Supplementary Information:**

The online version contains supplementary material available at 10.1186/s13049-023-01072-2.

## Background

Over the last few decades, the demand for emergency medical services (EMSs) has risen in many developed countries [1–3]. However, high non-conveyance rates and other measures suggest a possibly considerable over-triage in the dispatch of the EMS [4]. At the same time, a low fatality rate is reported for non-urgent dispatches, suggesting limited under-triage [4, 5]. Three independent expert boards have named the development of the EMS dispatch as one of the top emergency care research topics in the current era [6–8]. This highlights the need to evaluate the EMS data to enhance the consistency between the EMS dispatch and the acuteness of a patient’s condition and thus, to improve the usability of the EMS for time-critical patients [3].

Quick response from the emergency medical dispatcher (EMD) may reduce the first EMS unit’s time to reach the patient [8], but a problem may arise if the EMS units are dispatched urgently to a non-urgent incident and thus, are unable to respond immediately to other critical missions [9]. In truly time-critical situations such as cardiac arrests or strokes, rapid and correct dispatch is crucial [10–13]. This creates a situation where a certain amount of over-triage is necessary and acceptable to ensure that the patients receive immediate and proper response when needed. However, high rates of over-triage can be harmful for the EMS in numerous ways, leading to excessive costs, inappropriate use of resources [14], increased risk of ambulance crashes [15] and overfatigue of the EMS personnel [16]. To improve the quality of emergency dispatch, a new computer-assisted emergency dispatch system called Emergency Response Integrated Common Authorities (ERICA) was introduced in Finland in 2018 [12].

Since the introduction of ERICA, there have been no studies concerning the appropriateness of EMS dispatch. That is why this study´s first objective was to determine the consistency between the priority assessments of EMDs and of the EMS by measuring their over- and under-triage, efficiency, sensitivity, specificity and predictive values. All EMS dispatches made with ERICA in the Tampere University Hospital (Tays) area from 1 August 2021 to 31 August 2021 were analysed. The second objective of this study was to determine if the priority assessments consistency varies across the dispatch categories.

## Methods

### Setting

This study was conducted in the area of Tampere University Hospital, which covers 13,249 km^2^ of land and 2301 km^2^ of water, with a population of 527,478 [17, 18]. It has 38 advanced-care EMS units that are staffed by one nurse–paramedic and a paramedic or a firefighter. The EMSs are organised by the Tays Centre of Prehospital Emergency Care [19]. In Tays area all emergency calls are handled by the national Emergency Response Centre Agency (ERC Agency).

ERC Agency operates a nationwide, interconnected network of six Emergency Response Centres that receive all emergency calls and dispatch rescue services, EMSs, police and social services. Finland has only the official European emergency number (112) in use. In 2021, EMDs handled 2,754,870 emergency calls. ERC Agency dispatched 828,840 missions to the EMSs around the country [20]. Before 2018 the ERC Agency used a protocol similar to the Medical Priority Dispatch System to determine dispatch categories and priorities [11, 12] ERICA is even more rigid and computer-assisted system.

**Fig. 1:**
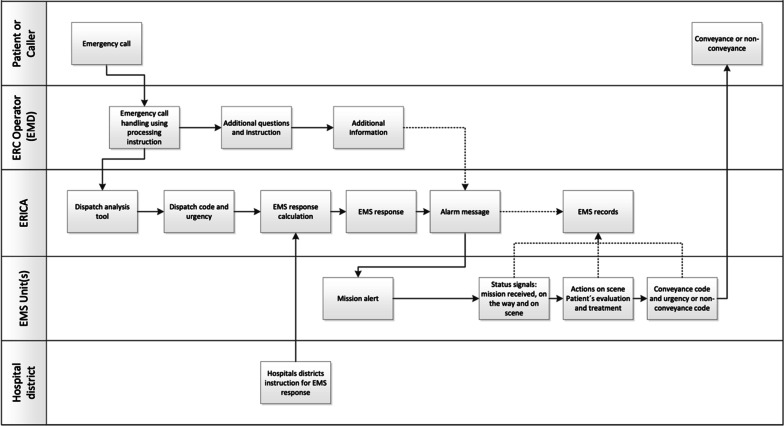
Medical emergency call process with the Emergency Response Integrated Common Authorities (ERICA)

With ERICA, the EMD uses processing instructions, and the nature of the emergency leads to a series of mandatory and non-mandatory follow-up questions. The dispatch code, which consists of the dispatch category and the dispatch priority, is automatically generated by the dispatch analysis tool [12]. Although ERICA is a nationwide system, all hospital districts can enter their own EMS response into the dispatch analysis tool (Fig. 1). This enables the use of the national dispatch criteria while considering the differences in the local EMS systems and responses around Finland.

EMSs have four dispatch priorities: (A) obvious or strongly suspected life-threatening incidents, (B) more stable urgent incidents, (C) semi-urgent incidents that require acute assessment and (D) non-urgent incidents. Priorities A and B both lead to an EMS dispatch with lights and siren (L&S), priority C requires that the patient be encountered within 30 min, and in priority D, within 2 h. In addition, there is a non-dispatch category for incidents that do not require an EMS response [21]. Besides the dispatch priority, the dispatch category also influences the EMS response. Not all A dispatches automatically involve rescue services or physician-staffed EMSs, but whether or not they will depend on the dispatch category.

### Study design

This prospective cohort study was conducted based on all EMS dispatches registered in the Tays area from 1 August 2021 to 31 August 2021. All EMS dispatches where EMS had confronted the patient were included. Dispatches where mission was cancelled, aborted, patient was not confronted, unit served as a first responder, or dispatch had unvalid data were excluded (n = 829). The EMS mission priority triaged by the EMD during the emergency call was compared with the EMS’s assessment of the patient’s priority on-scene. The EMS’s assessment of the priority was derived from the pre-set criteria (Table 1). For the comparison dispatch priorities A and B were deemed urgent, and priorities C and D were deemed non-urgent.

**Table 1:** Criteria for the priority assessment of the emergency medical services (EMS)

Urgent	Non-urgent
A paramedic’s ‘urgent’ priority assessment	A paramedic’s ‘non-urgent’ priority assessment
A/B dispatch and the patient was transported with A/B priority	C/D dispatch and the patient was transported with C/D priority or no transport
A/B dispatch and the patient had deceased	C/D dispatch and a deceased person
A/B dispatch and the patient received significant treatment^1^ (regardless of transportation)	A/B dispatch and the patient did not receive any significant treatment^1^ and was not transported with A/B priority
C/D dispatch and no transport but the patient received significant treatment^1^	
C/D dispatch and transportation with A/B priority	

A/B dispatch: EMS dispatch with lights and siren. C/D dispatch: EMS dispatch without lights and siren

^1^SpO2 < 95% for which the patient received bronchodilators; convulsion for which the patient received an anticonvulsant; allergic reaction for which the patient received epinephrine; any airway management, CPR or blood glucose < 4 for which the patient received IV glucose; SpCO > 5 for which the patient received oxygen; or overdosage or poisoning for which the patient received an antidote

The data were collected from the national ERC Agency system. Also, a copy of all EMS records from study period were collected from EMS service providers. The initial information included the incident address, time of the emergency call, mission alert time, dispatch category and priority, dispatched EMS unit, EMS time stamps (on the way, on-scene, patient encountered, beginning of the transportation, at the Emergency Department, patient signed over and mission completed), name of the Emergency Department if transported, transportation code and priority or non-transportation code. These records were then collected into a Microsoft Excel® table to which the research assistants manually inputted additional data from the patient’s EMS records (i.e., the patient’s medical history, vital signs, and any treatment received), which were validated by the authors TS and KK.

**Fig. 2:**
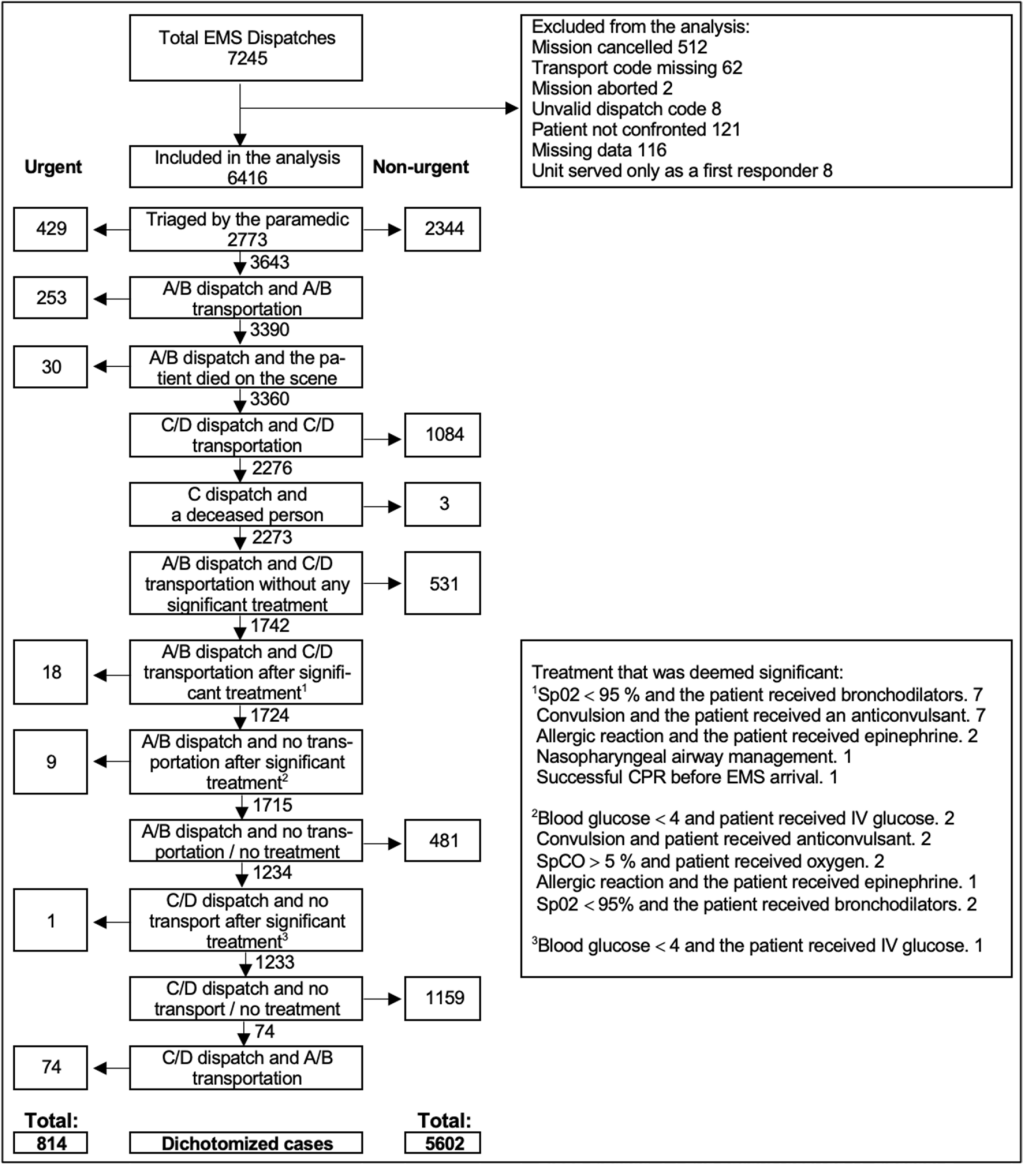
Dataflow and dichotomisation of the emergency medical services’ priority assessment

The data were also dichotomised into two groups according to EMS’s criteria (Table 1)—one where the situation was considered urgent and the other where the situation was considered non-urgent. This allowed comparison with other studies that used the same dichotomisation [22–24]. The dichotomisation was made in a specific order in which the paramedic’s assessment was primarily considered (Fig. 2).

The use of specific reference standards such as the National Advisory Committee for Aeronautics (NACA) scale [23–25] or the Medical Emergency Triage and Treatment System. Adult (METTS-A), which other authors had used [26, 27], was not possible in this study because the Finnish paramedics are not accustomed to routinely using any severity score. Although the NACA score was not used as a reference, the criteria of urgent and non-urgent patients were consistent with those in earlier studies [24]. The STROBE checklist was used to guide the manuscript preparation.

### Statistics

The test performance levels of the over- and under-triage levels, efficiency, sensitivity, specificity and predictive values were measured using the crosstabulation of true positive (TP), false positive (FP), true negative (TN) and false negative (FN) values and described as percentages with 95% confidence intervals. Crosstabulation and the chi-square test were used to analyse the consistency between the priority assessments. The Kruskal–Wallis H test was used to analyse the distribution of the dispatch consistency variable (four groups: TP, FP, TN and FN**)** among the 26 dispatch categories containing more than 50 dispatches to measure the variation of the consistency between the dispatch categories. The Bonferroni-corrected *p*-values were used in the post hoc test of pairwise comparison between dispatch categories. Percentages were calculated with Microsoft® Excel for Mac version 16.60 (Redmond, WA USA). Statistical analyses and crosstabulation were performed with IBM SPSS Statistics for MAC, version 27.0.1.0 (Armonk, NY USA), with a significance level of *p* < 0.05.

## Ethics

This was a prospective register-based study approved by the Tays research director (no. R21641). According to Finnish laws, the patient consent and the statement from the Ethics Committee were not needed, as this study was based on medical records and no interventions to patients were made.

## Results

There were 6416 EMS dispatches included in this study. The priority assessments of the EMD and the EMS were consistent in 72% (4637) of the dispatches. The priority was most consistent in *Cardiac arrest* (92%) and less consistent in *Unspecific symptoms* (12%). Table 2 presents the EMD dispatch priority distribution of all the dispatch categories that had more than 50 dispatches and their priority according to the EMS criteria.

**Table 2:** Differences between the dispatcher’s dispatch priority and the EMS priority assessment

Dispatch category (n)	EMD’s dispatch priority % (n)		EMS’s dispatch urgent		EMS’s dispatch non-urgent	
			EMS’s criteria priority % (n)			
	Urgent (A or B)	Non-urgent (C or D)	Urgent (TP)	Non-urgent (FP)	Non-urgent (TN)	Urgent (FN)
General weakness (984)	14 (139)	86 (845)	19 (26)	81 (113)	98 (827)	2 (18)
Fall (882)	18 (159)	82 (723)	21 (34)	79 (125)	98 (709)	2 (14)
Chest pain (631)	82 (516)	18 (115)	20 (105)	80 (411)	96 (110)	4 (5)
Breathing difficulty (407)	46 (186)	54 (221)	28 (53)	72 (133)	95 (211)	5 (10)
Psychiatric symptom^+^ (325)	N.A	100 (325)	N.A	N.A	94 (306)	6 (19)
Rhythm disorder (314)	26 (83)	74 (231)	19 (16)	81 (67)	99 (229)	1 (2)
Stroke (306)	82 (250)	18 (56)	26 (65)	74 (185)	96 (54)	4 (2)
Abdominal pain (286)	12 (34)	88 (252)	32 (11)	68 (23)	98 (247)	2 (5)
Hospital transport (253)	47 (120)	53 (133)	70 (84)	30 (36)	88 (117)	12 (16)
Poisoning (224)	38 (84)	62 (140)	50 (42)	50 (42)	91 (128)	9 (12)
Back pain (183)	5 (9)	95 (174)	22 (2)	78 (7)	98 (171)	2 (3)
Limb pain (144)	6 (9)	94 (135)	22 (2)	78 (7)	99 (134)	1 (1)
Nausea, diarrhoea, constipation (140)	2 (3)	98 (137)	67 (2)	33 (1)	98 (134)	2 (3)
Convulsion (137)	62 (85)	38 (52)	36 (31)	64 (54)	96 (50)	4 (2)
Headache (110)	42 (46)	58 (64)	15 (7)	85 (39)	97 (62)	3 (2)
Traffic accident, bicycle etc. (109)	40 (44)	60 (65)	32 (14)	68 (30)	100 (65)	0 (0)
Unconscious^+^ (99)	100 (99)	N.A	42 (42)	58 (57)	N.A	N.A
Traffic accident, small (97)	56 (54)	44 (43)	17 (9)	83 (45)	98 (42)	2 (1)
Impact/hit (76)	61 (46)	39 (30)	20 (9)	80 (37)	100 (30)	0 (0)
Unspecific symptoms^+^ (75)	100 (75)	N.A	12 (9)	88 (66)	N.A	N.A
Blood glucose problem (75)	25 (19)	75 (56)	32 (6)	68 (13)	95 (53)	5 (3)
Cut (67)	34 (23)	66 (44)	30 (7)	70 (16)	100 (44)	0 (0)
Allergic reaction (65)	69 (45)	31 (20)	20 (9)	80 (36)	100 (20)	0 (0)
Body pain (59)	7 (4)	93 (55)	0 (0)	100 (4)	93 (51)	7 (4)
Assault (54)	7 (4)	93 (50)	0 (0)	100 (4)	100 (50)	0 (0)
Cardiac arrest (51)	94 (48)	6 (3)	92 (44)	8 (4)	100 (3)	0 (0)

*TP* true positive, *FP* false positive, *TN* true negative, *FN* false negative, *N.A*. not available

^+^Only A/B or C/D dispatch was available

A or B: EMS dispatch with lights and siren. C or D: EMS dispatch without lights and siren

### EMD’s priority assessment compared with EMS’s priority assessment

There was a difference between the EMD and EMS priority assessments (*p* < 0.001) (Table 3). The EMD’s dispatch priority was urgent (A or B) in 2341 dispatches, but only 29% of those were urgent according to the EMS criteria. The EMD’s dispatch priority was non-urgent (C or D) in 4075 dispatches, and 97% of those were also non-urgent according to the EMS criteria. The overall efficiency was 72% (95% CI 71.2–73.4); sensitivity, 85% (95% CI 82.0–87.0); specificity, 71% (95% CI 69.3–71.1); positive predictive value (PPV), 29% (95% CI 27.5–31.2); and negative predictive value (NPV), 97% (95% CI 96.4–97.4).

**Table 3:** Dispatch priority and EMS priority assessment crosstabulation

EMD’s dispatch priority	EMS’s criteria priority			*p*-value
	Urgent % (n)	Non-urgent % (n)	Total % (n)	
				< 0.001
A (urgent)	55 (188)	45 (153)	5 (341)	Over-triage 71% (95% CI 68.8–72.5)
B (urgent)	25 (500)	75 (1500)	31 (2000)	
C (non-urgent)	4 (99)	96 (2161)	35 (2260)	Under-triage 3% (95% CI 2.6–3.6)
D (non-urgent)	1 (27)	99 (1788)	28 (1815)	
Total % (n)	13 (814)	87 (5602)	(6416)	

### Consistency between the priority assessments across the dispatch categories

The whole dataset included 55 dispatch categories, 26 of them containing more than 50 dispatches were compared (Table 4). There was a variation in the consistency between the priority assessments among dispatch categories (*p* = 0.000, Df. 25), for 95% confidence intervals, please see Additional file 1.

**Table 4:** Consistency between the priority assessments of the dispatcher and the EMS among the dispatch categories

Dispatch category (n)	Over-triage %	Under-triage %	Efficiency %	Sensitivity %	Specificity %	PPV%	NPV%
Body pain (59)	100	7	86	N.A	93	0	93
Assault (54)	100	0	93	N.A	93	0	100
Unspecific symptoms^+^ (75)	88	N.A	12	100	0	12	N.A
Headache (110)	85	3	63	78	61	15	97
Traffic accident, small (97)	83	2	53	90	48	17	98
General weakness (984)	81	2	87	59	88	19	98
Rhythm disorder (314)	81	1	78	89	77	19	99
Chest pain (631)	80	4	34	96	21	20	96
Impact/hit (76)	80	0	51	100	45	20	100
Allergic reaction (65)	80	0	45	100	36	20	100
Fall (882)	79	2	84	71	85	21	98
Back pain (183)	78	2	95	40	96	22	98
Limb pain (144)	78	1	94	67	95	22	99
Stroke (306)	74	4	39	97	23	26	96
Breathing difficulty (407)	72	4	65	84	61	29	96
Cut (67)	70	0	76	100	73	30	100
Abdominal pain (286)	68	2	90	69	92	32	98
Traffic accident, bicycle etc. (109)	68	0	73	100	68	32	100
Blood glucose problem (75)	68	5	79	67	80	32	95
Convulsion (137)	63	4	59	94	48	37	96
Unconscious^+^ (99)	58	N.A	42	100	0	42	N.A
Poisoning (224)	50	9	76	78	75	50	91
Nausea, diarrhoea, constipation (140)	33	2	97	40	99	67	98
Hospital transfer (253)	30	12	79	84	77	70	88
Cardiac arrest (51)	8	0	92	100	43	92	100
Psychiatric symptom^+^ (75)	N.A	6	94	0	100	N.A	94

Over-triage: 100-PPV; Under-triage: 100-NPV; Efficiency: ([TP + TN]/[TP + TN + FP + FN]) × 100; Sensitivity: (TP/[TP + FN]) × 100; Specificity: (TN/[TN + FP]) × 100; Positive predictive value (PPV): (TP/[TP + FP]) × 100;

Negative predictive value (NPV): (TN/(TN + FN]) × 100

*TP* true positive, *TN* true negative, *FP* false positive, *FN* false negative, *N.A*. not available

^+^Only A/B or C/D dispatch was available, which inhibited the calculation of certain variables

In the pairwise comparison of the TP, FP, TN and FN value distributions, all the dispatch categories had a difference (*p* < 0.05) with at least one of the other dispatch categories (Additional file 2).

The over-triage percentage significantly varied across the dispatch categories, with a range of 8–100%. Ten dispatch categories had an over-triage level of 80% or more, and 21 dispatch categories had an over-triage level of over 50%. *Unspecific symptoms* had the third highest over-triage percentage (88%) and currently does not even have the C or D dispatch priority available. The lowest over-triage percentage was in *Cardiac arrest. Hospital transfer* had the highest under-triage, though its priority was set by a physician instead of the EMD. The under-triage was 0% in six dispatch categories (Table 4).

Efficiency also had a high variation of 97–12%, with the highest being for *Nausea, diarrhoea and constipation.* Seven dispatch categories had a sensitivity of 100%, and only one dispatch category had a specificity of 100%. Sensitivity and specificity both ranged from 0 to 100%. Specificity was over 90% for *Psychiatric symptom*; *Nausea, diarrhoea and constipation*; *Back pain*; *Limb pain*; *Body pain*; *Assault*; and *Abdominal pain* (Table 4).

## Discussion

This study examined the consistency between the priority assessments of the EMD and the EMS and determined if such consistency varied among the dispatch categories. We found that the priority assessment of the EMD was much more overestimated than underestimated and had a higher sensitivity with moderate specificity when compared with the priority assessment of the EMS. Additionally, there were significant differences in the consistency between the EMD and EMS priority assessments across the dispatch categories.

In the non-urgent priority dispatches (C and D), there was minimal under-triage compared to the over-triage in the urgent priority dispatches (A and B). This phenomenon is supported by Dami et al. and Ball et al., who reported similar results [22, 23]. Also, the over- and under-triage, efficiency, sensitivity, specificity and predictive values of the new Finnish ERC Agency system are relatively consistent with those in the international research [22–24, 26–30].

A closer look reveals that the proportion of the urgent cases from the EMS perspective in the priority A dispatch was double that of the priority B dispatches. This indicates that the dispatch criteria can recognise the most urgent cases reasonably well and the non-urgent cases with high precision. The remarkably low over-triage for *Cardiac arrest* sheds light on this phenomenon; although all the patients suspected with cardiac arrest did not necessarily suffer from such, they most likely had a critical incident in the background that initially led to the suspicion. This issue is two-edged; for the EMS unit, the correct priority of the mission is paramount, and an incorrect dispatch category is not essential. Nevertheless, an incorrect dispatch category can lead to unnecessary dispatches for the physician-staffed EMS unit and the rescue services whose dispatches depend on the right dispatch category.

The low consistency in the priority B dispatches increased the over-triage and most likely emanated from the nature of priority B. Priority B had more cases that were unclear, and a dispatch with L&S was more of a precaution. Similar results were seen in an interview study; in situations where EMDs could not rule out an acute situation, they will send an ambulance just as a precaution [31]. The high over-triage level for *Unspecific symptoms* underlines this issue. *Unspecific symptoms* presents a unique dispatch criterion that complicates the priority assessment; despite the name of this category, the definition is that the caller is not with the patient or the patient cannot be contacted during the emergency call. For that reason, as a safety precaution, this category does not even have the C or D dispatch priority available. Nevertheless, *Unspecific symptoms* had the third highest over-triage percentage, suggesting that there is a need for a non-urgent dispatch option as well.

### Differences in the consistency between the EMD and EMS priority assessments across the dispatch categories

Although some dispatch categories had low frequencies and were thus not ideal for the analysis, there was still considerable variation in the results of the 26 dispatch categories that were analysed. This indicates differences in the overall validity of the dispatch criteria across the dispatch categories. The generally more non-critical symptoms (e.g., *Psychiatric symptom*; *Nausea, diarrhoea and constipation*; *Back pain*; *Limb pain* and *Body pain*) had the highest specificities, which indicates that the dispatch criteria are accurate when there is no presumptively high-risk patient. Controversially critical symptoms such as *Chest pain, Stroke* and *Unconsciousness* had low efficiency. These incidents require high sensitivity to ensure that all critically ill patients will receive rapid and adequate dispatch; but at some point, oversensitivity eventually decreases specificity and efficiency.

The PPV was 50% or less in 22 dispatch categories. This is alarming, since it means that a guess could be as accurate as the current triage of the urgent incidents, and thus, it eliminates the benefits of a telephone triage. The ultimate intention of a telephone triage is to ensure the responsiveness and efficiency of the EMS process [32]. It is not achieved if the dispatch criteria are not efficient, which requires adequate sensitivity and specificity. On the other hand, high NPV levels indicate that the dispatch system is safe for the patient.

More time should be taken to clarify the priority of the situation when the EMD does not have a reasonable suspicion of a life-threatening situation. Nowadays, the Finnish ERC Agency uses solely time intervals as the quality indicators. That can create a situation for the ERC Agency personnel where a fast dispatch is considered more important than an accurate priority assessment. Simultaneously, it is important to remember that evaluating the priority of the patient via telephone is not the same as doing so face to face with professional expertise and instruments [33].

Many other factors influence emergency call handling, such as interpersonal or communication variables [34–36]. Machine learning and video calls are new tools that have been introduced to support the EMD’s decision-making process [37–40]. Further research is required to illustrate what factors cause inaccurate urgent dispatch in certain dispatch categories. Criteria leading to priority B should be investigated in all dispatch categories to evaluate what causes significant over-triage.

## Strengths and limitations

The most valuable strength of this study was its precise dichotomisation of the priority levels from the EMS’s perspective with numerous criteria. This is because comparison of the mere dispatch and transportation codes or non-transport rates within the dispatch categories would have led to wider bias. The short inclusion period and the size of the dataset also led to some limitations. Because rarer dispatch categories had only one or a few cases, no conclusion could be made regarding the appropriateness of the dispatch criteria in those categories. In addition, due to the regional data collection, the general applicability of the results to other areas is uncertain. On the other hand, the smaller sample size made it possible for us to evaluate the data more profoundly. Had we collected national data or had a longer inclusion period, the large data size would not have allowed us to manually screen all the EMS records to sort out what kind of treatment the patients had received from the EMS.

Finnish EMS system has one unique feature; the transport priority is also used to describe the usability of the ambulance for an intercurrent dispatch. For that reason, the conveyance priority can be A or B also for non-medical reasons. This can cause a minimal risk of bias in case where non-urgent dispatch was considered false negative because of urgent transport code for non-medical reason. An additionally registered conveyance priority does not necessarily mean that there was an actual L&S conveyance; it could also have been merely a precaution from the paramedic. For the duration of this study, the EMS personnel were advised that they could document their priority assessment of the situation when the patient was confronted, but this was not mandatory. This limited the possibility of a bias caused by an inaccurate transport priority.

## Conclusion

Of all the urgent EMD dispatches, 71% were not urgent according to the EMS criteria, which decreased the EMS usability. The non-urgent dispatches were recognised with high accuracy; therefore, it is safe to dispatch non-emergency units or to keep the non-urgent missions on hold. Ten dispatch categories that had the over-triage level of 80% or more require immediate and critical appraisal of the dispatch criteria. Measures must be taken to ensure efficient use of the EMS resources in the future.

## Supplementary Information

**Additional file 1:** Measured test performance levels and their 95% confidence intervals. Test performance levels of over- and under-triage, efficiency, sensitivity, specificity, and predictive values with their 95% confidence intervals among the 26 dispatch categories.

**Additional file 2:** All Kruskall–Wallis H test pairwise comparisons that had a significant difference. All pairwise comparisons that had a significant difference, based on the Kruskall–Wallis H test of TP/FP/TN/FN distribution among the dispatch codes and their significance levels (Bonferroni-corrected).

## Data Availability

The data that support the findings of this study are available from Tays but with restrictions, as they were used under license and so are not publicly available. However, the data are available from the authors upon reasonable request and with the permission of the Tays research director.

## Abbreviations

EMD: Emergency medical dispatcher
EMS: Emergency medical service
ERC Agency: National Emergency Response Centre Agency
ERICA: Emergency Response Integrated Common Authorities
FN: False negative
FP: False positive
L&S: Lights and Siren
NACA: National Advisory Committee for Aeronautics
METTS-A: Medical Emergency Triage and Treatment System-Adult
NPV: Negative predictive value
PPV: Positive predictive value
Tays: Tampere University Hospital
TN: True negative
TP: True positive
