# Supplementary material for: Differences between the dispatch priority assessments of emergency medical dispatchers and emergency medical services: a prospective register-based study in Finland
Source: Scand J Trauma Resusc Emerg Med. 2023 Feb 16;31:8. doi: 10.1186/s13049-023-01072-2 (PMC9936687; doi:10.1186/s13049-023-01072-2)
Supplement: Supplementary file 1 — Additional file 1: Measured test performance levels and their 95% confidence intervals. Test performance levels of over- and under-triage, efficiency, sensitivity, specificity, and predictive values with their 95% confidence intervals among the 26 dispatch categories. [file 13049_2023_1072_MOESM1_ESM.pdf]

Additional file 1 Measured test performance levels and their 95% confidence intervals (CI).

	Over-triage		Under-triage		Efficiency		Sensitivity		Specificity		PPV		NPV	
**Dispatch category**	**%**	**95% CI**	**%**	**95% CI**	**%**	**95% CI**	**%**	**95% CI**	**%**	**95% CI**	**%**	**95% CI**	**%**	**95% CI**
General weakness	**81**	78.9 – 83.7	**2.1**	1.2 – 3.0	**87**	84.6 – 88.8	**59**	56.0 – 62.2	**88**	85.9 – 90.0	**19**	16.3 – 21.1	**98**	97.0 – 98.8
Fall	**79**	75.9 – 81.3	**1.9**	1.0 – 2.8	**84**	81.8 – 86.6	**71**	67.8 – 73.8	**85**	82.7 – 87.4	**21**	18.7 – 24.1	**98**	97.2 – 99.0
Chest pain	**80**	76.5 – 82.8	**4.3**	2.8 – 5.9	**34**	30.4 – 37.8	**95**	93.8 – 97.1	**21**	17.9 – 24.3	**20**	17.2 – 23.5	**96**	94.1 – 97.2
Breathing difficulty	**72**	67.1 – 75.9	**4.5**	2.5 – 6.5	**65**	60.2 – 69.5	**84**	80.6 – 87.7	**61**	56.6 – 66.1	**28**	24.1 – 32.9	**95**	93.5 – 97.5
Psychiatric symptom	**N.A.**	0.0 – 0.0	**5.8**	3.3 – 8.4	**94**	91.6 – 96.7	**0**	0.0 – 0.0	**100**	100.0 – 100.0		0.0 – 0.0	**94**	91.6 – 96.7
Rhythm disorder	**81**	76.4 – 85.1	**0.9**	0.0 – 1.9	**78**	73.4 – 82.6	**89**	85.4 – 92.4	**77**	72.7 – 82.0	**19**	14.9 – 23.6	**99**	98.1 – 100.0
Stroke	**74**	69.1 – 78.9	**3.6**	1.5 – 5.7	**39**	33.4 – 44.4	**97**	95.1 – 989	**23**	17.9 – 27.3	**26**	21.1 – 30.9	**96**	94.3 – 98.5
Abdominal pain	**68**	62.2 – 73.1	**2.0**	0.4 – 3.6	**90**	86.8 – 93.7	**69**	63.4 – 74.1	**91**	88.2 – 94.7	**32**	26.9 – 37.8	**98**	96.4 – 99.6
Hospital transport	**30**	24.4 – 35.6	**12.0**	8.0 – 16.0	**79**	74.5 – 84.4	**84**	79.5 – 88.5	**76**	71.2 – 81.7	**70**	64.4 – 75.6	**88**	84.0 – 92.0
Poisoning	**50**	43.5 – 56.5	**8.6**	4.9 – 12.2	**76**	70.3 – 81.5	**78**	72.3 – 83.2	**75**	69.6 – 80.9	**50**	43.5 – 56.5	**91**	87.8 – 95.1
Back pain	**78**	71.8 – 83.8	**1.7**	0.0 – 3.6	**95**	91.2 – 97.8	**40**	32.9 – 47.1	**96**	93.3 – 98.9	**22**	16.2 – 28.2	**98**	96.4 – 100.0
Limb pain	**78**	71.0 – 84.6	**0.7**	0.0 – 2.1	**94**	90.7 – 98.2	**67**	59.0 – 74.4	**95**	91.5 – 98.6	**22**	15.4 – 29.0	**99**	97.9 – 100.0
Nausea, diarrhoea, constipation	**33**	25.5 – 41.1	**2.2**	0.0 – 4.6	**97**	94.4 – 99.9	**40**	31.9 – 48.1	**99**	97.8 – 100.0	**67**	58.9 – 74.5	**98**	95.4 – 100.0
Convulsion	**64**	55.5 – 71.6	**3.8**	0.6 – 7.1	**59**	50.9 – 67.4	**94**	89.9 – 97.9	**48**	39.7 – 56.4	**36**	28.4 – 44.5	**96**	92.9 – 99.4
Headache	**85**	78.1 – 91.5	**3.1**	0.0 – 6.4	**63**	53.7 – 71.8	**78**	70.0 – 85.5	**61**	52.3 – 70.5	**15**	8.5 – 21.9	**97**	93.6 – 100.0
Traffic accident, bicycle etc.	**68**	59.4 – 76.9	**0.0**	0.0 – 0.0	**72**	64.1 – 80.9	**100**	100.0 – 100.0	**68**	59.7 – 77.1	**32**	23.1 – 40.6	**100**	100.0 – 100.0
Unconscious	**58**	47.8 – 67.3	**N.A.**	0.0 – 0.0	**42**	32.7 – 52.2	**100**	100.0 – 100.0	**0**	0.0 – 0.0	**42**	32.7 – 52.2		0.0 – 0.0
Traffic accident, small	**83**	75.9 – 90.7	**2.3**	0.0 – 5.3	**53**	42.6 – 62.5	**90**	84.0 – 96.0	**48**	38.3 – 58.2	**17**	9.3 – 24.1	**98**	94.7 – 100.0
Impact/hit	**80**	71.5 – 89.4	**0.0**	0.0 – 0.0	**51**	40.1 – 62.6	**100**	100.0 – 100.0	**45**	33.6 – 56.0	**20**	10.6 – 28.5	**100**	100.0 – 100.0
Unspecific symptoms	**88**	80.6 – 95.4	**N.A.**	0.0 – 0.0	**12**	4.6 – 19.4	**100**	100.0 – 100.0	**0**	0.0 – 0.0	**12**	4.6 – 19.4		0.0 – 0.0
Blood glucose problem	**68**	57.9 – 78.9	**5.4**	0.3 – 10.5	**79**	69.4 – 87.9	**67**	56.0 – 77.3	**80**	71.3 – 89.3	**32**	21.1 – 42.1	**95**	89.5 – 99.7
Cut	**70**	58.5 – 80.6	**0.0**	0.0 – 0.0	**76**	65.9 – 86.3	**100**	100.0 – 100.0	**73**	62.7 – 83.9	**30**	19.4 – 41.5	**100**	100.0 – 100.0
Allergic reaction	**80**	70.3 – 89.7	**0.0**	0.0 – 0.0	**45**	32.5 – 56.7	**100**	100.0 – 100.0	**36**	24.1 – 47.4	**20**	10.3 – 29.7	**100**	100.0 – 100.0
Body pain	**100**	100.0 – 100.0	**7.3**	0.6 – 13.9	**86**	77.7 – 95.2	**0**	0.0 – 0.0	**93**	86.1 – 99.4	**0**	0.0 – 0.0	**93**	86.1 – 99.4
Assault	**100**	100.0 – 100.0	**0.0**	0.0 – 0.0	**93**	85.6 – 99.6		0.0 – 0.0	**93**	85.6 – 99.6	**0**	0.0 – 0.0	**100**	100.0 – 100.0
Cardiac arrest	**8**	0.7 – 15.9	**0.0**	0.0 – 0.0	**92**	84.8 – 99.5	**100**	100.0 – 100.0	**43**	29.3 – 564	**92**	84.1 – 99.3	**100**	100.0 – 100.0

PPV: Positive predictive value; NPV: Negative predictive value; N.A.: Not available
