# Supplementary material for: Differences between the dispatch priority assessments of emergency medical dispatchers and emergency medical services: a prospective register-based study in Finland
Source: Scand J Trauma Resusc Emerg Med. 2023 Feb 16;31:8. doi: 10.1186/s13049-023-01072-2 (PMC9936687; doi:10.1186/s13049-023-01072-2)
Supplement: Supplementary file 2 — Additional file 2: All Kruskall–Wallis H test pairwise comparisons that had a significant difference. All pairwise comparisons that had a significant difference, based on the Kruskall–Wallis H test of TP/FP/TN/FN distribution among the dispatch codes and their significance levels (Bonferroni-corrected). [file 13049_2023_1072_MOESM2_ESM.pdf]

Additional file 2 All Kruskall–Wallis H test pairwise comparisons that had a significant difference.

Dispatch category 1 – Dispatch category 2	Sig.	Adj. Sig. [a]
Allergic reaction – Abdominal pain	0.000	0.000
Allergic reaction – Assault	0.000	0.000
Allergic reaction – Back pain	0.000	0.000
Allergic reaction – Blood glucose problem	0.000	0.000
Allergic reaction – Body pain	0.000	0.000
Allergic reaction – Fall	0.000	0.000
Allergic reaction – General weakness	0.000	0.000
Allergic reaction – Limb pain	0.000	0.000
Allergic reaction – Nausea, diarrhoea, constipation	0.000	0.000
Allergic reaction – Poisoning	0.000	0.008
Allergic reaction – Psychiatric symptom	0.000	0.000
Allergic reaction – Rhythm disorder	0.000	0.000
Blood glucose Problem – Psychiatric symptom	0.000	0.012
Breathing difficulty – Abdominal pain	0.000	0.000
Breathing difficulty – Assault	0.000	0.029
Breathing difficulty – Back pain	0.000	0.000
Breathing difficulty – Body pain	0.000	0.000
Breathing difficulty – Fall	0.000	0.000
Breathing difficulty – General weakness	0.000	0.000
Breathing difficulty – Limb pain	0.000	0.000
Breathing difficulty – Nausea, diarrhoea, constipation	0.000	0.000
Breathing difficulty – Psychiatric symptom	0.000	0.000
Breathing difficulty – Rhythm disorder	0.000	0.000
Cardiac arrest – Abdominal pain	0.000	0.000
Cardiac arrest – Allergic reaction	0.000	0.000
Cardiac arrest – Assault	0.000	0.000
Cardiac arrest – Back pain	0.000	0.000
Cardiac arrest – Blood glucose problem	0.000	0.000
Cardiac arrest – Body pain	0.000	0.000
Cardiac arrest – Breathing difficulty	0.000	0.000
Cardiac arrest – Chest pain	0.000	0.000
Cardiac arrest – Convulsion	0.000	0.000
Cardiac arrest – Cut	0.000	0.000
Cardiac arrest – Fall	0.000	0.000
Cardiac arrest – General weakness	0.000	0.000
Cardiac arrest – Headache	0.000	0.000
Cardiac arrest – Hospital transfer	0.000	0.000
Cardiac arrest – Impact/hit	0.000	0.000
Cardiac arrest – Limb pain	0.000	0.000
Cardiac arrest – Nausea, diarrhoea, constipation	0.000	0.000
Cardiac arrest – Poisoning	0.000	0.000
Cardiac arrest – Psychiatric symptom	0.000	0.000
Cardiac arrest – Rhythm disorder	0.000	0.000
Cardiac arrest – Stroke	0.000	0.001
Cardiac arrest – Traffic accident, bicycle	0.000	0.000
Cardiac arrest – Traffic accident, small	0.000	0.000
Chest pain – Abdominal pain	0.000	0.000
Chest pain – Assault	0.000	0.000
Chest pain – Back pain	0.000	0.000
Chest pain – Blood glucose problem	0.000	0.000
Chest pain – Body pain	0.000	0.000
Chest pain – Breathing difficulty	0.000	0.000

Chest pain – Cut	0.000	0.000
Chest pain – Fall	0.000	0.000
Chest pain – General weakness	0.000	0.000
Chest pain – Headache	0.000	0.000
Chest pain – Hospital transfer	0.000	0.000
Chest pain – Limb pain	0.000	0.000
Chest pain – Nausea, diarrhoea, constipation	0.000	0.000
Chest pain – Poisoning	0.000	0.000
Chest pain – Psychiatric symptom	0.000	0.000
Chest pain – Rhythm disorder	0.000	0.000
Chest pain – Traffic accident, bicycle	0.000	0.000
Chest pain – Traffic accident, small	0.000	0.001
Convulsion – Abdominal pain	0.000	0.000
Convulsion – Assault	0.000	0.000
Convulsion – Back pain	0.000	0.000
Convulsion – Blood glucose problem	0.000	0.000
Convulsion – Body pain	0.000	0.000
Convulsion – Fall	0.000	0.000
Convulsion – General weakness	0.000	0.000
Convulsion – Limb pain	0.000	0.000
Convulsion – Nausea, diarrhoea, constipation	0.000	0.000
Convulsion – Poisoning	0.000	0.001
Convulsion – Psychiatric symptom	0.000	0.000
Convulsion – Rhythm disorder	0.000	0.000
Cut – Back pain	0.000	0.007
Cut – Limb pain	0.000	0.037
Cut – Nausea, diarrhoea, constipation	0.000	0.003
Cut – Psychiatric symptom	0.000	0.000
Fall – Psychiatric symptom	0.000	0.000
General weakness – Psychiatric symptom	0.000	0.000
Headache – Abdominal pain	0.000	0.000
Headache – Back pain	0.000	0.000
Headache – Body pain	0.000	0.002
Headache – Fall	0.000	0.006
Headache – General weakness	0.000	0.000
Headache – Limb pain	0.000	0.000
Headache – Nausea, diarrhoea, constipation	0.000	0.000
Headache – Psychiatric symptom	0.000	0.000
Hospital transfer – Abdominal pain	0.000	0.000
Hospital transfer – Assault	0.000	0.002
Hospital transfer – Back pain	0.000	0.000
Hospital transfer – Blood glucose problem	0.000	0.012
Hospital transfer – Body pain	0.000	0.000
Hospital transfer – Fall	0.000	0.000
Hospital transfer – General weakness	0.000	0.000
Hospital transfer – Limb pain	0.000	0.000
Hospital transfer – Nausea, diarrhoea, constipation	0.000	0.000
Hospital transfer – Psychiatric symptom	0.000	0.000
Hospital transfer – Rhythm disorder	0.000	0.000
Impact/hit – Abdominal pain	0.000	0.000
Impact/hit – Assault	0.000	0.001
Impact/hit – Back pain	0.000	0.000
Impact/hit – Blood glucose problem	0.000	0.005
Impact/hit – Body pain	0.000	0.000
Impact/hit – Fall	0.000	0.000

Impact/hit – General weakness	0.000	0.000
Impact/hit – Limb pain	0.000	0.000
Impact/hit – Nausea, diarrhoea, constipation	0.000	0.000
Impact/hit – Psychiatric symptom	0.000	0.000
Impact/hit – Rhythm disorder	0.000	0.000
Poisoning – Abdominal pain	0.000	0.000
Poisoning – Back pain	0.000	0.000
Poisoning – Body pain	0.000	0.001
Poisoning – Fall	0.000	0.000
Poisoning – General weakness	0.000	0.000
Poisoning – Limb pain	0.000	0.000
Poisoning – Nausea, diarrhoea, constipation	0.000	0.000
Poisoning – Psychiatric symptom	0.000	0.000
Rhythm disorder – Back pain	0.000	0.002
Rhythm disorder – General weakness	0.000	0.036
Rhythm disorder – Limb pain	0.000	0.044
Rhythm disorder – Nausea, diarrhoea, constipation	0.000	0.001
Rhythm disorder – Psychiatric symptom	0.000	0.000
Stroke – Abdominal pain	0.000	0.000
Stroke – Assault	0.000	0.000
Stroke – Back pain	0.000	0.000
Stroke – Blood glucose problem	0.000	0.000
Stroke – Body pain	0.000	0.000
Stroke – Breathing difficulty	0.000	0.000
Stroke – Cut	0.000	0.000
Stroke – Fall	0.000	0.000
Stroke – General weakness	0.000	0.000
Stroke – Headache	0.000	0.000
Stroke – Limb pain	0.000	0.000
Stroke – Nausea, diarrhoea, constipation	0.000	0.000
Stroke – Poisoning	0.000	0.000
Stroke – Psychiatric symptom	0.000	0.000
Stroke – Rhythm disorder	0.000	0.000
Stroke – Traffic accident, bicycle	0.000	0.000
Stroke – Traffic accident, small	0.000	0.001
Stroke –Hospital transfer	0.000	0.000
Traffic accident, bicycle – Abdominal pain	0.000	0.000
Traffic accident, bicycle – Back pain	0.000	0.000
Traffic accident, bicycle – Body pain	0.000	0.001
Traffic accident, bicycle – Fall	0.000	0.001
Traffic accident, bicycle – General weakness	0.000	0.000
Traffic accident, bicycle – Limb pain	0.000	0.000
Traffic accident, bicycle – Nausea, diarrhoea, constipation	0.000	0.000
Traffic accident, bicycle – Psychiatric symptom	0.000	0.000
Traffic accident, small – Abdominal pain	0.000	0.000
Traffic accident, small – Assault	0.000	0.004
Traffic accident, small – Back pain	0.000	0.000
Traffic accident, small – Blood glucose problem	0.000	0.047
Traffic accident, small – Body pain	0.000	0.000
Traffic accident, small – Fall	0.000	0.000
Traffic accident, small – General weakness	0.000	0.000
Traffic accident, small – Limb pain	0.000	0.000
Traffic accident, small – Nausea, diarrhoea, constipation	0.000	0.000
Traffic accident, small – Psychiatric symptom	0.000	0.000
Traffic accident, small – Rhythm disorder	0.000	0.001

Unconsciousness – Abdominal pain	0.000	0.000
Unconsciousness – Allergic reaction	0.000	0.001
Unconsciousness – Assault	0.000	0.000
Unconsciousness – Back pain	0.000	0.000
Unconsciousness – Blood glucose problem	0.000	0.000
Unconsciousness – Body pain	0.000	0.000
Unconsciousness – Breathing difficulty	0.000	0.000
Unconsciousness – Chest pain	0.000	0.000
Unconsciousness – Cut	0.000	0.000
Unconsciousness – Fall	0.000	0.000
Unconsciousness – General weakness	0.000	0.000
Unconsciousness – Headache	0.000	0.000
Unconsciousness – Hospital transfer	0.000	0.000
Unconsciousness – Impact/hit	0.000	0.000
Unconsciousness – Limb pain	0.000	0.000
Unconsciousness – Nausea, diarrhoea, constipation	0.000	0.000
Unconsciousness – Poisoning	0.000	0.000
Unconsciousness – Psychiatric symptom	0.000	0.000
Unconsciousness – Rhythm disorder	0.000	0.000
Unconsciousness – Stroke	0.000	0.009
Unconsciousness – Traffic accident, bicycle	0.000	0.000
Unconsciousness – Traffic accident, small	0.000	0.000
Unconsciousness –Convulsion	0.000	0.000
Unspecific symptom – Abdominal pain	0.000	0.000
Unspecific symptom – Assault	0.000	0.000
Unspecific symptom – Back pain	0.000	0.000
Unspecific symptom – Blood glucose problem	0.000	0.000
Unspecific symptom – Body pain	0.000	0.000
Unspecific symptom – Breathing difficulty	0.000	0.000
Unspecific symptom – Convulsion	0.000	0.006
Unspecific symptom – Cut	0.000	0.000
Unspecific symptom – Fall	0.000	0.000
Unspecific symptom – General weakness	0.000	0.000
Unspecific symptom – Headache	0.000	0.000
Unspecific symptom – Hospital transfer	0.000	0.000
Unspecific symptom – Impact/hit	0.000	0.006
Unspecific symptom – Limb pain	0.000	0.000
Unspecific symptom – Nausea, diarroea, constipation	0.000	0.000
Unspecific symptom – Poisoning	0.000	0.000
Unspecific symptom – Psychiatric symptom	0.000	0.000
Unspecific symptom – Rhythm disorder	0.000	0.000
Unspecific symptom – Traffic accident small	0.000	0.000
Unspecific symptom – Traffic accident, bicycle	0.000	0.000

Each row tests the null hypothesis that the Sample 1 and Sample 2 distributions are the same. Asymptotic significances (two-sided tests) are displayed. The significance level was 0.050.

[a] Significance values have been adjusted by the Bonferroni correction for multiple tests.
